# Cancer Patient Advocacy in the Postoperative Intensive Care Unit: The Experience of Nurses and the Voice of Older Adult Patients

**DOI:** 10.3390/healthcare14121618

**Published:** 2026-06-09

**Authors:** Sara Morais Pires, Idalina Gomes

**Affiliations:** Nursing Research Innovation and Development Centre of Lisbon (CIDNUR), School of Nursing, Universidade de Lisboa, 1600-096 Lisbon, Portugal; idgomes@enfermagem.ulisboa.pt

**Keywords:** patient advocacy, grounded theory, geriatric oncology, perioperative care, intensive care unit

## Abstract

**Background/Objectives**: Older adults with cancer in the postoperative environment face complex vulnerability, exacerbated by the frailty of ageing and the aggressiveness of surgical treatment. In this highly demanding context, nurses play a crucial role as patient advocates. However, there is a knowledge gap regarding how advocacy is perceived and experienced by the nurse-patient dyad. This qualitative study aims to explain the practice of advocacy by comparing the perspectives of nurses and patients in order to construct part of a substantive theory on the subject. **Methods**: The Grounded Theory methodological approach was adopted. The sample included 6 specialist nurses and 10 older cancer patients from the ICU. Data collection consisted of participant observation and semi-structured interviews with both groups of participants. The analysis followed the constant comparison method, using MAXQDA software (version 24.10.0; VERBI Software; Berlin, Germany), allowing for the systematic and comparative identification of codes and categories from the two data sources. **Results**: The core process, defined as The Advocacy-Adjustment Dyad, reveals how older adult cancer patients navigate critical care through a symbiotic interplay of coping and support. Patients autonomously deploy Internal Adjustment mechanisms namely, Shielding the Emotional Self, to mitigate disease stress. Concurrently, the nurse operationalizes the Dynamic Expert Nurse Advocacy Cycle through a Therapeutic Alliance that prioritizes the patient’s best interest, integrates the Family as an anchor, and ensures a meaningful understanding of information. This dyadic interaction transforms the ICU from a purely technological setting into a therapeutic space, ensuring the Preservation of Personhood and the safeguarding of the patient’s self-determination. **Conclusions**: This study is pioneering in integrating the patient’s voice into the construction of a theory on advocacy in critical care, demonstrating that its effectiveness is a process of mutual interaction and not merely a unilateral function of the nurse. The findings emphasise the need to actively include the patient’s perspective in training and policy, offering valuable implications for optimizing older adult-centered care.

## 1. Introduction

The global demographic landscape is undergoing a profound transformation, with population ageing emerging as one of the greatest strategic challenges for public health today. According to the World Health Organisation, by 2030, one in six people is expected to be aged 60 or over, a phenomenon driven by increased life expectancy and declining birth rates [1]. This reality is particularly critical in the European context and, specifically, in Portugal. Currently, the country has one of the most ageing demographic structures in the European Union, with around 25% of its population aged 65 or over. This scenario requires urgent reorganisation and more efficient management of the resources allocated to care provision [2,3].

Aging stands as the primary driver in the development of oncological diseases, with cancer incidence increasing exponentially with age. Often, cancer diagnosis in older adults does not occur in isolation, but rather in a context of multimorbidity, where cardiovascular, metabolic, and neurodegenerative diseases complicate therapeutic decisions and impact prognosis [4,5]. Cancer is intrinsically linked to increased frailty, which in turn significantly raises the risk of treatment-related toxicity, hospitalization, and mortality [6]. This state of complex vulnerability, which compromises the patient’s physiological reserve, has been shown to be an independent predictor of post-operative morbidity, functional decline and reduced tolerance to treatment [6].

In this context, the older adult population presents a profile of high vulnerability, with functional deficits concentrated in mobility, cognition, and the performance of activities of daily living. Recent research identifies very advanced age (≥85 years), low educational attainment, and depressive emotional state as critical predictors of greater functional impairment [7,8]. This clinical frailty means that a significant percentage of older adult cancer patients have severe deficits in self-care, requiring full compensation strategies on the part of healthcare teams to ensure continuity of care and quality of life [7,8,9].

Given this complexity, perioperative nursing plays a central role in ensuring safe, person-centered care. This specialty encompasses structured interventions in the pre-, intra-, and postoperative phases, aiming at rigorous risk assessment and optimization of clinical outcomes [10,11]. In the postoperative period, the clinical focus shifts to continuous monitoring, pain management, prevention of complications, and functional rehabilitation, which are fundamental elements in mitigating the impact of surgery on the autonomy of older adult cancer patients [10,12].

In addition, evidence-based research highlights the imperative of integrating patient advocacy during this phase, recognizing the decisive role of these professionals in safeguarding individuals with diminished self-determination and complex vulnerability [13,14]. Central to professional nursing is the role of the advocate: a steadfast commitment to safeguarding patient rights, autonomy, and safety, especially when navigating the complexities of oncological surgery in older adults [14,15]. Acting as the patient’s “advocate” in the postoperative period theoretically involves conflict mediation, supporting informed decision-making, and ensuring that the dignity of the older adults is preserved in the face of the technological dominance of the healthcare system [13,16].

This need for protection is further reinforced by the evolution of the concept of advocacy, which has shifted from a paternalistic approach to a paradigm centered on promoting the patient’s autonomy and self-determination. Underpinning this role is an inalienable ethical commitment, transcending mere technical competence to become a cornerstone of patient safety [17]. As such, this moral duty is essential for safeguarding the dignity of the individual, particularly in highly technical settings such as Intensive Care Units. In these contexts, the qualitative literature demonstrates that the patient’s extreme vulnerability requires the nurse to act as the guardian of their fundamental rights, preventing the risk of dehumanization in care [17,18].

In intensive care and perioperative settings, advocacy is crucial to ensuring that patients in a state of extreme vulnerability receive the information necessary for informed choices, mitigating the risks of hasty decisions in crisis environments [19,20]. However, the effectiveness of this intervention is often constrained by structural barriers, like excessive workload and professional burnout, as critical obstacles that can silence this moral duty and compromise vigilance regarding patient dignity [21,22].

Conversely, strengthening professional autonomy and recognising the patient as a social and family member emerge as key factors in reversing this situation [23]. The evidence suggests that by adopting this holistic approach, nursing not only protects individuals from clinical risks but also enhances their satisfaction and empowers them to take charge of their own care, ensuring that safety is integrated with respect for the patient’s personal narrative and life plans [23,24].

Despite the clinical and ethical salience of patient advocacy, contemporary literature remains predominantly descriptive, focusing on theoretical conceptualizations or isolating the nursing perspective within general medical-surgical wards [14,15,20,22,25]. This unidirectional focus introduces a critical empirical blind spot, as it relegates the immediate perspective of care recipients to the periphery and fails to capture how advocacy is operationally co-constructed within highly acute, technologically dense environments. To rigorously evaluate how advocacy safeguards patient safety and dignity, research must transcend this fragmented framework. It is imperative to critically examine the interactional synergy between the professional maneuvers of nurses and the acute, lived experiences of older adult patients within the critical crucible of the post-operative Intensive Care Unit (ICU).

Currently, a critical empirical gap exists regarding how patient advocacy is dynamically co-constructed within acute, postoperative ICU environments.

Guided by this imperative, and building upon a previous conceptualization that mapped the operational dynamics of advocacy from the professional standpoint [13], the present study aims to explore the practice of patient advocacy within the specific context of the Postoperative Intensive Care Unit (ICU), focusing on the surgical journey of older oncology patients. While our earlier work explored the nurses’ experiences [13], a comprehensive understanding of this phenomenon requires capturing the perspectives of all core participants. Therefore, this research shifts the analytical lens entirely to the patients’ viewpoint, seeking to generate an emergent conceptual model based on the actions, interactions, and lived experiences of older adult patients. Ultimately, it aims to identify how advocacy can effectively safeguard the dignity and self-determination of this vulnerable population, bridging the gap between high-technology care and the subjective needs of the older adult population.

## 2. Materials and Methods

Rooted in symbolic interactionism and the interpretive paradigm, Grounded Theory (GT) was employed as the methodological framework for this research. Originally conceptualized by Glaser and Strauss (1967), this inductive, iterative methodology enables the generation of theory intrinsically grounded in empirical evidence [26]. This study opted for the systematized approach of Strauss and Corbin (2015) [26,27]. The adoption of the “Straussian” line is justified by its structured coding and constant comparison framework, which allows for accurate mapping of care trajectories and patient responses. By focusing specifically on the nurse-patient dyad, this methodology provided the analytical rigor and plasticity needed to capture the multidimensionality of advocacy, effectively integrating the lived experiences and vulnerabilities of older adult oncological patients with the ethical and clinical responses of healthcare professionals.

### 2.1. Researcher Positioning

In alignment with the interpretive underpinnings of this study, the theorization process emerged from the dialectical engagement between the researcher and the participants. The lead investigator, a specialist in Medical-Surgical and Perioperative Nursing with 18 years of clinical expertise, utilized this background to enhance theoretical sensitivity. To mitigate potential biases, it is critical to note that the researcher was an “outsider” to the specific postoperative ICU where the study took place, having no clinical or administrative ties to the local healthcare team. Furthermore, explicit reflexivity procedures were operationally enforced to manage subjectivity: the primary investigator maintained a detailed reflexive journal throughout the fieldwork to log immediate insights, and weekly peer-debriefing sessions were conducted within the research team to cross-examine emerging categories against raw transcripts. This systematic interaction ensures that the resulting substantive theory is a grounded reflection of the intricate human, ethical, and clinical interactions that characterize the oncological perioperative experience.

### 2.2. Recruitment

The study was conducted in Portugal, a country with approximately 10 million inhabitants and a mixed healthcare system that integrates public and private providers into the National Health Service. Recruitment took place at an internationally renowned Oncology Institution (anonymous: H1), specifically in its Postoperative Intensive Care Unit (ICU). This study is part of a larger research project investigating the perioperative period with a total sample of 45 participants.

This unit specializes in the perioperative management of complex cancer patients, with 8 beds and a nurse-to-patient ratio of 1:2. This staffing model is crucial due to the high clinical severity and the need for advanced monitoring and constant procedures. The care environment is marked by high clinical and ethical complexity, given the critical nature of cancer diagnoses.

The care environment is characterised by a high workload, imposed by the clinical and ethical pressure inherent in critical cancer diagnoses. However, the organisational culture of the institution (H1) acts as a facilitator of the nurse’s role as patient advocate by actively promoting interprofessional synergy through transparent ethical discourse among medical and nursing staff. This structural and ethical support creates the necessary foundation for defending the rights and dignity of patients in vulnerable situations.

Regarding the selection process, the research began with intentional sampling, progressively evolving into theoretical sampling, in accordance with the iterative nature of GT. The field phase was preceded by a project presentation session at the unit, conducted by the principal investigator. This allowed for initial contact with potential participants, preliminary observations of the context, and detailed clarification of methodological and ethical issues, ensuring the transparency of the data collection process. The semi-structured interview guides were theoretically grounded in literature on patient advocacy and acute care vulnerability. Content validity was verified by an expert panel (two PhD researchers and two ICU specialists), and the guides were pilot-tested with one nurse and one patient to confirm semantic clarity, and no changes were required. Inclusion criteria were defined to target perioperative oncology nurses possessing at least 6 years of direct experience in the surgical management of cancer patients. With regard to patients, individuals over 65 years of age with cancer in the postoperative phase were included. Participation was strictly voluntary, and all individuals, both nurses and patients, provided informed consent after a detailed explanation of the study’s objectives. Based strictly on these selection criteria and driven by the sequential needs of the theoretical sampling process, a total of 6 nurses and 10 patients were approached and consecutively included (*n* = 16).

The cohort ultimately comprised those who actively consented to share their narratives, ensuring that the empirical data reflected a diverse range of perspectives and experiences inherent to the nurse-patient dyad in the oncological postoperative context.

### 2.3. Data Collection

Anchored in the perspective of symbolic interactionism, this study posits that social reality and professional practices are co-constructed through ongoing human interaction, communication, and the sharing of meanings among the actors involved. In accordance with GT methodology, data collection was based on a triangulation of methods that included non-participant observation, in-depth interviews, and document analysis.

Field observations took place at the H1 oncology institution, centering on the delivery of perioperative care to the geriatric oncological population. The records were made in a field diary and subsequently digitised and analysed, which made it possible to identify the characteristics of the environment, the actions of the healthcare teams and the patterns of interaction that shape care practices, capturing non-verbal dynamics and contextual interactions during clinical rounds.

At the same time, in-depth interviews were conducted with both perioperative oncology nurses, recognised for their extensive clinical experience, and patients in the postoperative period, in order to capture the direct experience of the phenomenon under investigation. The interviews, lasting approximately 30 to 60 min, were recorded on audio and transcribed in full. The interview guides evolved dynamically early findings from initial analyses drove the refinement of prompts in subsequent interviews to capture deeper dimensions of the participants’ experiences. Data analysis was carried out iteratively and concurrently with data collection, allowing for the continuous refinement of emerging categories and the triangulation of findings between different groups of participants. In addition, an analysis of institutional and regulatory documents was carried out, including hospital protocols, clinical guidelines and patient safety policies, which provided the necessary structural and normative context to contrast with the empirical narratives. The data collection process was enriched by an iterative triangulation of sources, encompassing semi-structured interviews, field notes from clinical observations, and extensive analytical memos. These were further complemented by diagrammatic mapping and a reflexivity journal, ensuring a robust audit trail and fostering high levels of theoretical sensitivity.

### 2.4. Data Analysis

The sample size (6 nurses, 10 patients) was determined concurrently with data analysis through theoretical sampling. Initial participant narratives provided foundational codes regarding the phenomenon. Following the constant comparative method, subsequent participants were purposefully sampled to challenge, refine, and densify the emerging conceptual properties, ensuring sufficient analytical depth for this specific intensive care context.

Data analysis began simultaneously with data collection, respecting the principles of simultaneity and iterativity of Grounded Theory. The analytical process was developed in sequential but interconnected phases, aiming to integrate the perspectives of nurses and the experiences shared by patients in the post-operative period. The management, organisation and coding of all empirical material were supported by MAXQDA version 24.10.0 qualitative analysis software.

**Figure 1:**
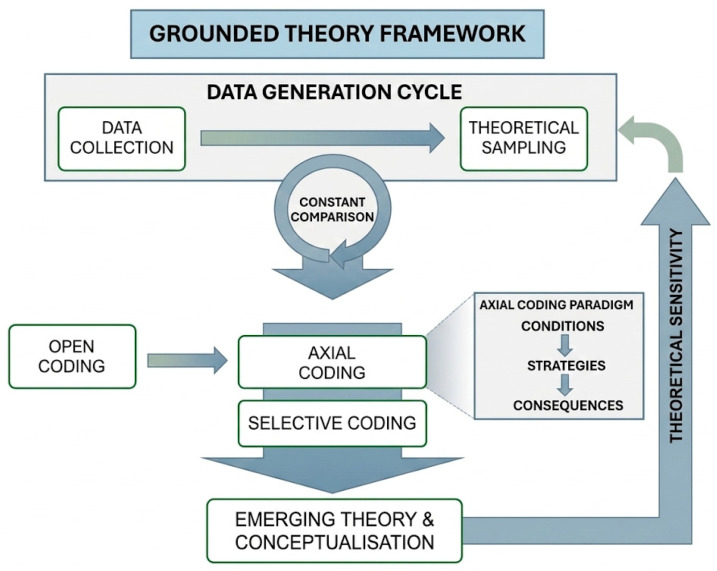
Methodological flowchart of the Grounded Theory Framework.

The analytical process began with Open Coding, an exhaustive breakdown of data aimed at identifying key concepts, actions, and interactions within perioperative care for older patients with cancer. Through a meticulous process of line-by-line coding for interview transcripts and incident-by-incident coding for observational field notes, we generated a robust set of descriptive and in vivo codes. Subsequently, Axial Coding was employed to regroup and correlate emerging categories. By applying the Coding Paradigm, systematically linking conditions, the central phenomenon, and action strategies, as illustrated in Figure 1, we were able to map the intricate interconnections that either facilitate or hinder patient advocacy, thereby deepening the analytical and interpretative density of our findings.

Throughout the research process, the memo-writing technique was used. These records served to document theoretical reflections, preliminary interpretations, and analytical questions that emerged from direct confrontation with the data, guiding the subsequent stages of theoretical sampling and the consolidation of the emerging theory, ensuring that it reflected the complexity of the ethical and clinical interactions observed in the field.

Throughout the research, memo-writing served as an essential analytical tool, documenting methodological reflections, evolving interpretations, and inquiries that informed the iterative stages of theoretical sampling. Coding procedures were carefully tailored to the data source. Including the systematic analysis of institutional documentation such as nursing care plans, clinical protocols, and institutional guidelines for postoperative cancer care. These documents served as an objective baseline for data triangulation; by cross-examining them with participant narratives, we were able to identify the gaps and alignments between formal institutional prescriptions and the lived clinical realities of nurse advocacy, directly refining and elevating the conceptual precision and abstraction of the emerging categories. The interpretation of the findings was framed by symbolic interactionism, emphasizing how nurses and patients co-construct meanings and how these shared interpretations shape advocacy practices within the oncological setting.

Theoretical saturation was achieved after concurrent analysis of the 6th nurse and 10th patient transcripts, alongside field notes. At this stage, during active research team meetings, comparative analysis of consecutive narratives yielded redundant interactional patterns rather than new conceptual properties, dimensions, or relationships. This was operationally verified when the independent coding tracks of the co-authors ceased to produce any new discrete codes for three consecutive transcripts. This point of conceptual stability confirmed that the boundaries of the emergent categories were robustly defined, ensuring methodological soundness given the complexity of oncological intensive care. The entire comparative analysis process and its traceability were managed using MAXQDA software.

### 2.5. Rigor

The scientific basis of this study rested on the rigorous application of the assumptions of credibility, transferability, confirmability, and dependency, which are fundamental pillars of GT. The research was conducted with a commitment to absolute fidelity to the data, guided by contemporary methodological guidelines that favour analytical density. The credibility of the theoretical model was achieved through a strategy of triangulation of sources, harmonising information extracted from direct observations in the field, analysis of institutional documents and exhaustive interviews with two distinct groups: nurses and patients. This constant comparative method ensured that the theory remained intrinsically linked to empirical reality, while the systematic use of in vivo codes ensured that the participants’ voices and language were preserved in the conceptual structure.

Beyond memoing, two operational reflexivity procedures were enforced: first, a dual-perspective analytical grid was maintained during coding to cross-reference raw codes with field notes, verifying if interpretations were driven by the participants’ voices rather than clinical habits; second, weekly peer-debriefing sessions were conducted where co-authors acted as ‘critical challengers’, randomly auditing transcript lines against the assigned codes to actively disrupt subjective assumptions. In addition, external validation by a second independent researcher allowed for a critical review of the categories and their interrelationships, lending objectivity and robustness to the findings.

In terms of transferability, the study offers a detailed characterization of both the oncological setting and the profile of the 16 participants (6 nursing professionals and 10 postoperative patients). This level of descriptive detail allows the scientific community to assess the resonance of this theory in clinical contexts with similar characteristics. Sampling was guided by a logic of maximum variation, deliberately selecting profiles that would enrich the emerging categories. Finally, confirmability was consolidated through a meticulous audit trail, supported by memos that trace the entire path from raw data to final theoretical abstraction, ensuring the traceability and integrity of the research.

### 2.6. Ethical Considerations

To ensure the research adhered to internationally recognized ethical standards, the study was designed and conducted in strict accordance with the principles of the Declaration of Helsinki and the CIOMS guidelines, fundamentally upholding the core tenets of patient autonomy, transparency, beneficence, and justice. The protocol obtained formal approval from the Ethics Committee and the Board of Directors of the hospital (H1), ensuring authorization from the departments involved for its execution, approved by the Ethics Committee (20240716.06 of 24 September 2024) for studies involving humans. At the heart of ethical conduct was the recognition of vulnerability as a universal human condition, which carries emotional and psychological risks inherent in the voluntary sharing of life experiences. In this sense, strict procedures of clarification and free and informed consent were implemented for the 6 nurses and 10 patients, ensuring absolute confidentiality and the right to withdraw from participation at any time.

The approach to older adult cancer patients in the postoperative period required special ethical care, based on the principles of beneficence and non-maleficence. To strictly safeguard patient autonomy within this vulnerable state, informed consent was operationalized as a dynamic, ongoing process rather than a single bureaucratic event. Beyond the initial signature, the principal investigator performed a continuous verification of consent before and during the dialogue, explicitly reminding patients of their absolute right to pause, skip questions, or withdraw at any moment without any impact on their clinical care. The researcher remained highly sensitive to non-verbal cues of distress or physical fatigue, actively offering breaks to ensure participation remained strictly voluntary. Furthermore, In preparing the informational documents and in oral interaction, a deliberate decision was made not to mention the cancer diagnosis, focusing on the fact that the person was ill or had undergone surgery. This decision was based on the assumption that a cancer diagnosis makes older people more vulnerable and can increase multidimensional suffering. In addition, it was recognized that communicating the diagnosis is the sole responsibility of the medical team and not the researcher, and that disclosure should be selective and respectful of the patient’s individual and sociocultural characteristics.

To ensure anonymity, all data were coded and stored on secure platforms, in accordance with the General Data Protection Regulation (GDPR), ensuring that the methodological rigor of GT coexisted with the highest respect for the psychosocial integrity of the subjects involved.

## 3. Results

To establish the foundation for our qualitative analysis, we first synthesized the sociodemographic and professional profiles of the participating nurses. The systematization of this data, drawn from specialists in postoperative cancer care, is crucial for establishing the profile of the interviewees and contextualising their views on the role of the nurse as an advocate for older adult with cancer. The detailed presentation of indicators such as age, gender, length of professional practice (particularly in an oncology context) and specialist training constitutes a pillar of methodological transparency, serving as the basis for a robust interpretation of the results and emerging categories (Table 1).

Continuing with the description of the study cohort, the following section presents a profile of the group of patients who took part in the research. Analysing individual profiles is essential for contextualising the health trajectories and subjective experiences gathered during the interviews. This section details the sociodemographic and clinical variables, namely age, gender, occupation before retirement and the specific cancer diagnosis. Understanding these characteristics allows for a deeper understanding of each participant’s advocacy needs and coping mechanisms in the face of cancer, lending greater rigour and transparency to the interpretation of the qualitative data presented below (Table 2).

While data from nursing professionals provided essential context regarding the operational conditions and strategies of care, this analysis intentionally prioritizes the rich data that emerged from the patient interviews. This methodological choice is grounded in the epistemological principles of Grounded Theory and Symbolic Interactionism, recognizing the patient as the primary agent experiencing postoperative vulnerability in the first person. This is not merely aimed at complementing different viewpoints, but rather at triangulating perspectives in order to provide conceptual depth to the phenomenon of patient advocacy in a perioperative intensive care unit. By integrating the lived experience and the subjective adjustment process of the person receiving care, the theory extends beyond the clinical dimension, capturing the interactivity of care in the perioperative period. Thus, the following explanation details the trajectory of the coding process, from initial fragmentation to axial integration, grounding the emergence of the theoretical model in the direct voices of the protagonists and ensuring that the theory remains strictly grounded in the empirical reality of this group.

The presentation of these findings mirrors the procedural and inductive logic inherent to Grounded Theory, wherein conceptualization is continuously refined through iterative, interconnected analytical cycles. The inductive analysis followed the systematic coding steps of Grounded Theory (open, axial, and selective coding). To ensure full transparency and an auditable trail of our conceptual development, the complete analytical trajectory from initial open codes to the final selective categories is detailed in Table 3. This taxonomy forms the basis of the intermediate process model depicting the older adult’s experience with cancer surgery, thereby ensuring methodological rigor and grounding the emergent theory firmly in empirical evidence.

### 3.1. Navigating Fragility

The selective category “Navigating Fragility” describes the state of extreme vulnerability experienced by older adults patients in the ICU, where the “self” feels threatened by the oncological crisis. This process is characterised by physical and psychological fragmentation imposed by technology and dependency, and is exacerbated by comorbidities and post-operative complications that undermine biological resilience. Navigating this scenario of uncertainty is permeated by profound suffering and pain, fuelled by the fear of metastases and the direct confrontation with finitude in a near-death experience, making fragility the central condition that justifies the protective intervention of advocacy. While ‘Navigating Fragility’ defines this passive, ontological state of exposure to threat, it is conceptually distinct from ‘Shielding the Self’, which represents the active psychological and behavioral strategies subsequently deployed by the patient to cope with this vulnerability and preserve their identity.

“*At first, it was almost a cataclysm in my life.*” (H1, Participant 6, Patient, ICU).

“*I felt awful, but that’s just how my body reacted, as I’ve already said… I felt myself slipping away; one good thing about crossing over is that you don’t think about your family, you don’t do anything…*” (H1, Participant 1, Patient, ICU).

### 3.2. Engaging Family in Advocacy

“Engaging Family in Advocacy” positions the family as a vital support system and an extension of the older adults. While nurses leverage family unity and affection to foster resilience, the model identifies a frequent delegation of decisions to relatives due to the patient’s fragility. However, the emergence of family pressure regarding surgery requires nurses to act as mediators, ensuring that family involvement sustains the patient’s dignity without compromising their individual autonomy.

“*My daughter knows everything. And she’s been pulling the strings with everything we’ve done so far, as I often say. She has a file containing all the medical records I have here, along with my entire medical history, all the tests, and everything I need to do.*” (H1, Participant 3, Patient, ICU).

“*We’re very close. And my daughter lives in Spain and came straight over here. That’s how it is. She helps me. She helps me a lot.*” (H1, Participant 4, Patient, ICU).

### 3.3. Managing Vulnerability Through the Therapeutic Alliance

“Managing Vulnerability through the Therapeutic Alliance” defines the relational bond as a core clinical intervention. Patients identify valuing nursing care viewed as “half the cure” and humanizing care as essential to their recovery. By being present and listening, nurses actively manage the patient’s vulnerability, creating a climate of safety. This alliance, reinforced by the patient’s trust in the doctor, functions as a protective network that stabilizes the older adults individual’s emotional state and ensures their dignity is preserved throughout the perioperative process.

“*It’s half the cure. It’s half the cure. If the patient is confident, that’s half the cure… This is very important, and that ‘half the cure’ is the confidence we have, the support.*” (H1, Participant 1, Patient, ICU).

“*It’s not just their competence, professionalism and empathy; the way they work with cancer patients is incredible, because this place is all about oncology, isn’t it? But when you walk into the building, you feel a sense of calm, it’s quite something. Impressive.*” (H1, Participant 9, Patient, ICU).

“*…And she’s a nurse who helps me enjoy life more and gives me more support.*” (H1, Participant 3, Patient, ICU).

### 3.4. Preserving Personhood Through the Advocacy

The core process “Preserving Personhood” represents the continuous integration of professional advocacy and the patient’s internal adjustment. The data demonstrate that patients maintain their identity by mobilizing faith and religious beliefs and fostering optimism and resilience. This internal strengthening is anchored in the future, specifically by focusing on grandchildren and recreational activities, which act as relational links to their life outside the ICU. Finally, the acceptance of the reality of the illness serves as the cognitive foundation that allows the older adults patient to transition from a state of vulnerability to one of integrated endurance, ensuring their essence is preserved throughout the surgical trajectory.

“*I asked God to give me some of his patience. So that I can face things. So that I can cope. And so that things get off to a good start. Things are going well.*” (H1, Participant 1, Patient, ICU).

“*Nobody hid anything. There’s no point in hiding anything. That’s how we talk. We’ve always been there. I’ve always forged ahead. With great determination.*” (H1, Participant 4, Patient, ICU).

“*For the first two days, yes… because I live for my grandson! And then… I felt really down, but then I also thought that to help him, I need to be well too*” (H1, Participant 2, Patient, ICU).

### 3.5. Synchronizing Advocacy with the Patient’s Self-Determination

“Synchronizing Advocacy” describes the process of aligning nursing interventions with the older adults patient’s autonomy and cognitive needs. The data reveal a proactive search for agency, characterized by a persistent wanting to be informed and well-informed about the clinical status and the therapeutic plan. This synchronization is operationalized through the constant clarification and justification of nursing and medical procedures, which allows the patient to construct an accurate recollection of events amidst the disorientation of the ICU. In some instances, patients manifest an overemphasis on details as a strategy to regain a sense of control over their body and the environment. By facilitating this navigating of the healthcare system, the nurse acts as a mediator who ensures that the patient’s self-determination is respected, transforming the surgical trajectory into a transparent and shared experience.

“*At the nursing consultation, they give us this information, they give us a booklet, they give us contact details for during the week, a mobile number for during the week, and a mobile number for the weekend, so that we can get in touch if we have any questions or to send them a photo.*” (H1, Participant 5, Patient, ICU).

“*Whenever I ask for information, whoever I ask, everyone is helpful. They explain how things are, how things were, what’s going to happen, and what has already happened.*” (H1, Participant 2, Patient, ICU).

“*What I want is an explanation every now and then; I actually check up on everything the nurses do, and the doctors too, and anything I notice that might be wrong, it just doesn’t sit right with me. I want to know more about this situation.*” (H1, Participant 3, Patient, ICU).

“*I already knew I had a tiny lump. And other doctors had said it wasn’t a big deal. However, (…), after I developed lung cancer, they thought* [bowel cancer with lung metastases]*… The doctor thought we needed to find out what was going on with the lump. So we started having tests. CT scans and PET scans, and then a biopsy was requested to find out. From then on, we realised straight away that we had to do something.*” (H1, Participant 5, Patient, ICU).

### 3.6. Shielding the Self

This category describes the psychological mechanisms used by patients to modulate the emotional impact of their diagnosis. A striking finding across the data was the mostly omission of the word “cancer” by patients throughout the postoperative trajectory, where the pathology was systematically left unnamed to maintain distance from its stigma. This process is reinforced by the construction of therapeutic optimism, with patients prioritizing minor clinical improvements to overshadow the gravity of their prognosis. Additionally, the denial or downplaying of the seriousness of both the surgery and the underlying disease acts as a collective cognitive buffer, allowing the older adults to maintain emotional stability while navigating the invasive reality of the ICU.

“*And then I spoke to this guy… And I said-Look, mate, my friend was heading for the middle. And he said to me, Do you know what that is? I told him: Let’s not stop. Let’s move forward faster now.*” (H1, Participant 2, Patient, ICU).

“*All in all, the situation hasn’t been too bad. We go through some rough patches, some worse ones, and some that are absolutely dreadful at times, don’t we? But, at the moment, the situation is, for me, practically normal—good, yes, good—I said good—in all the sessions I’ve been involved in as a patient.*” (H1, Participant 3, Patient, ICU).

**Figure 2:**
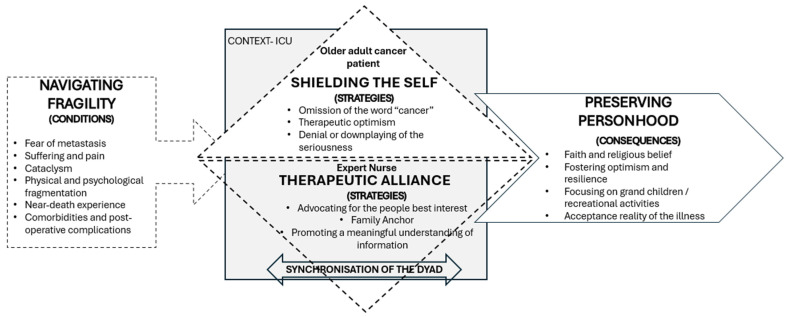
The advocacy-adjustment dyad.

The following scheme (Figure 2), illustrates the theoretical model “Navigating Fragility and Preserving Personhood”, which conceptualizes the Advocacy-Adjustment Dyad as the central process of care. This processual model describes the surgical trajectory of the older adult patients in the ICU, structured as a directional process flow that moves chronologically from left to right through conditions, strategies, and consequences. The trajectory emerges from a baseline phase of Navigating Fragility, which establishes the antecedent clinical and existential conditions. This state of extreme vulnerability is triggered by suffering and pain, the fear of metastasis, and the clinical impact of comorbidities and post-operative complications. This oncological cataclysm leads to a state of physical and psychological fragmentation, often exacerbated by a near-death experience during the critical period. As indicated by the directional arrow, this overwhelming fragility is the baseline condition that directly drives the process into the ICU context, activating a dual strategic framework known as the Advocacy-Adjustment Dyad. This central phenomenon operates through two parallel layers within the ICU. On the internal layer, the patient protects themselves by Shielding the Self (omitting the word “cancer”, therapeutic optimism, and downplaying the illness). Simultaneously, on the external layer, the nurse builds the Therapeutic Alliance (advocating for the patient’s best interests, using the family as an anchor, and clarifying information). At the base, the bidirectional arrow of Synchronisation of the Dyad shows the continuous feedback loop between nursing advocacy and patient autonomy. Finally, this dynamic synergy flows into Preserving Personhood as the ultimate consequence, where faith, resilience, family links, and acceptance successfully maintain the patient’s identity amidst the technological environment of the ICU.

## 4. Discussion

The experience of older adult cancer patients in a post-operative intensive care setting reveals a journey marked by a profound ontological crisis, encapsulated in the concept of “Navigating Fragility”. This state of vulnerability, exacerbated by the threat to the integrity of the “self” caused by the cancer crisis, is not limited to physical decline or post-operative complications. However, it reflects the experience of psychosomatic disintegration in the face of finitude. In this scenario, technology, whilst essential for survival, paradoxically acts as a factor that underscores dependence, forcing the patient into a constant confrontation with their own mortality.

Current literature emphasises that managing this vulnerability depends on the effectiveness of perioperative support interventions. In this context, successful outcomes in geriatric oncology are determined by the integration of support strategies that go beyond the technical dimension, mitigating the disruptive impact of hospitalisation on the cognitive resilience of older adults. The absence of these strategies compromises the patient’s emotional stability and coping ability, making the nurse’s role as an advocate a protective barrier against the fragmentation of the self [28].

Within the category of “Engaging Family in Advocacy”, the family emerges as an extension of the older patient’s agency. However, this integration introduces a critical tension that is often oversimplified in perioperative literature. While our previous work [14] conceptualized family integration as a standard attribute of advocacy, our current findings reveal a more problematic dynamic in the ICU setting: biological frailty frequently forces a premature delegation of decisions, creating a risk of family pressure overriding the patient’s actual wishes. Therefore, the nurse’s role as an intermediary is not merely to “align” support [14], but to actively police the boundaries of autonomy. As synthesized in contemporary literature, health advocacy is fundamentally an ethical process of managing interpersonal vulnerabilities, where nurses must proactively navigate conflicting values between family members and the healthcare team to ensure the preservation of the patient’s individual self-determination [29].

This nuanced perspective contrasts with traditional family-centered care frameworks, which often assume a natural harmony between the family’s protective instincts and the patient’s autonomy, failing to account for the silent erasure of the older adult’s voice during critical care dependency. This complex interpersonal dynamic is heavily aligned with published findings which demonstrate, that when family choices regarding life-supportive therapies clash with clinical perceptions of futility, intense ethical conflicts emerge among ICU nurses. These conflicts, driven by concerns over patient suffering, can inadvertently disrupt the quality of family-centered care and distance the healthcare team from the family unit [30].

The category “Managing Vulnerability through the Therapeutic Alliance” defines the therapeutic relationship as a foundational clinical intervention, in which patients regard nursing care as “half the cure”. This perception highlights that presence and active listening are essential for humanising the experience in the ICU and creating a sense of emotional safety. This understanding that presence and active listening are fundamental to creating a climate of emotional safety is supported by evidence that patient advocacy is intrinsically linked to nurses’ professional values; the more firmly established these values are, the more effective patient advocacy becomes, thereby strengthening mutual trust [31]. These findings corroborate the data from this study by suggesting that the therapeutic alliance acts as a protective network that stabilises the emotional state of older adult cancer patients. Thus, technical competence and empathy act as antidotes against disorientation and the fragmentation of the “self”, ensuring that dignity is maintained throughout the perioperative journey [31,32].

Concerning the “Preserving Personhood through the advocacy” category, this describes the maintenance of the older person’s identity through the mobilisation of resources such as faith and optimism. This process of internal adjustment is enhanced by the nurse’s advocacy, by recognising the patient as a unique individual, the nurse protects their dignity in a critical care setting. According to a recent literature review with meta-analysis, supportive interventions such as nurse coaching in oncology are fundamental to reducing existential stress and promoting resilience, enabling the patient to maintain their sense of self despite the crisis imposed by the illness [33]. The imperative to protect the patient’s identity is further validated by recent evidence, from an umbrella review, which underscores that psychological and emotional needs remain the most frequently unmet domains, emphasizing that nursing advocacy must proactively bridge these gaps to prevent the loss of identity and quality of life in cancer survivors [34].

With regard to “Synchronizing Advocacy with Patients Self-Determination”, this reflects the alignment of nursing interventions with the older adults person’s proactive pursuit of autonomy. The data indicate that the persistent desire for information and control over the treatment plan enables the patient to rebuild their sense of autonomy within the invasive environment of the ICU. This finding is corroborated by evidence, that associates individualised care in perioperative contexts with the nurse’s ability to recognise the patient’s needs and involve them in decision-making [35].

In this process, the nurse acts as a mediator, transforming the surgical journey into a transparent and shared experience [35]. The literature emphasises that a pro-advocacy attitude on the part of healthcare professionals is predictive of safer, person-centred care [13]. Thus, the constant clarification of information and the justification of procedures are not mere formalities, but advocacy strategies that support the self-determination and empowerment of older cancer patients, mitigating the disorientation typical of critical care [13,32,34].

Building upon this, the category “Shielding the Self” reflects the self-protection mechanism whereby older adults downplay their diagnosis in order to preserve their emotional well-being in the face of the disruption caused by the illness. This “shield” functions as an adaptive internal coping strategy that must be respected by the nurse’s advocacy, avoiding clinical confrontations that may overwhelm the patient’s capacity to process information. Some authors maintain that spiritual care and respect for the patient’s beliefs are fundamental to reducing the impact of existential stress, allowing the patient to process the reality of cancer at their own pace [34]. Thus, by integrating emotional and spiritual support, the nurse validates the older adults person’s defence mechanisms, ensuring that clinical practice does not undermine the patient’s sense of identity, but rather provides a safe environment for coming to terms with the diagnosis [32,35].

In conclusion, this study offers an original conceptualization of the Dynamic Expert Nurse Advocacy Cycle by shifting the focus from a static view of advocacy to a fluid, interconnected feedback loop within the Advocacy-Adjustment Dyad. Theoretically, this framework provides a nuanced understanding of how professional advocacy interventions and patient-driven coping mechanisms continuously reinforce one another within the critical care environment. Practically, these insights provide a vital clinical roadmap in geriatric oncology. Rather than managing technology in a detached manner, critical care units must actively mitigate the older patient’s psychosomatic fragmentation, transforming technical competence into an ethical shield that preserves autonomy and identity during an acute cancer crisis.

## 5. Strengths and Limitations

This study’s primary strength is rooted in its methodological rigor and unique focus on the high-acuity perioperative setting. The application of Grounded Theory has proven instrumental in generating a substantive theory that captures the nuances of patient advocacy. While this model is an emergent construct, representing a preliminary, rather than a definitive, explanation of the phenomenon, it provides a crucial analytical lens for understanding the dynamics of care for older adults in oncological surgery. A significant strength is the triangulation of perspectives, incorporating data from both nurses (*n* = 6) and patients (*n* = 10). This dual approach provides a comprehensive understanding of advocacy, capturing not only the professional intentionality and ethical decision-making but also the direct impact and perception of those receiving care. Additionally, conducting the study in an International Reference Oncology Unit provides great credibility and depth to the data, ensuring that insights into managing existential stress, spirituality, and institutional barriers were collected in a setting of clinical excellence. This focus on a highly critical population—older adults with cancer in an Intensive Care Unit—fills a significant knowledge gap and holds transferability potential for similar high-complexity healthcare settings.

The limitations of this study are consistent with the intrinsic nature of qualitative inquiry. Primarily, the findings possess context-specific transferability, as they are derived from an in-depth analysis of a single setting: a Perioperative Intensive Care Unit within a reference oncological institution. While the sample size (*n* = 16) was robust enough to achieve theoretical saturation, the hallmark of a grounded theory approach, the resulting model is inevitably influenced by the site’s unique cultural environment, specialized resources, and organizational protocols.

Consequently, the transferability of this substantive theory to other healthcare settings should be assessed through conceptual analogy. This requires acknowledging that the identified barriers and facilitators to patient advocacy are deeply embedded in the specific organizational fabric of the study site. It is essential to underscore that the objective of this research is not statistical generalization, but rather, it seeks to generate and validate an emergent theory that provides a nuanced explanation of the complexities of advocacy throughout this specific clinical trajectory.

## 6. Conclusions

The findings of this study have significant implications for perioperative and intensive care nursing, suggesting that advocacy must be transitioned from an intuitive moral duty to a structured clinical intervention. First, the Dynamic Expert Nurse Advocacy Cycle provides a pedagogical framework for training programs, enabling less experienced nurses to develop the “theoretical sensitivity” required to navigate complex ethical dilemmas. Second, healthcare organizations should implement formal protocols that prioritize the “dual approach”—relational and informational—ensuring that time for active listening and family mediation is integrated into the clinical workflow, rather than treated as a secondary task. Finally, the evidence regarding “Complex Vulnerability” calls for the development of multidisciplinary care pathways in geriatric oncology that formally recognize the nurse as an ethical steward. By addressing the identified organizational barriers, institutions can foster an environment where nursing advocacy acts as a sustainable care, effectively reducing the risk of depersonalization and enhancing the self-determination of older adult cancer patients.

## Figures and Tables

**Table 1:** Socio-professional profile of the participating expert nurses (*n* = 6).

Characteristics	*n*
Gender	
Male	2
Female	4
Age	
25–30	0
31–40	2
41–50	4
Years of Professional Experience	
1–10	0
11–20	3
21–30	3
Postgraduate Education	
Master	3
Nursing Specialization	4

**Table 2:** Sociodemographic and clinical characteristics of the participants.

Gender	Older Adult Participant (*n* = 10)
Male	5
Female	5
Age	
65–70	3
71–75	4
76–80	1
81–85	2
Pre-retirement occupation	
Administrative Assistant	2
Secondary School Teacher	2
Business Owner	3
Security forces	1
Telecommunications technician	1
Bank employee	1
Cancer Diagnosis	
Bowel cancer	3
Gastric cancer	1
Pancreatic cancer	2
Esophageal cancer	1
Thyroid cancer	1
Breast cancer	2

**Table 3:** Examples of the Evolution of Coding: From Open to Selective (Grounded Theory).

Open Coding	Axial Coding	Selective Coding
Fear of metastasis Suffering and pain Cataclysm Physical and psychological fragmentation Near-death experience Comorbidities and post-operative complications	Vulnerability and Existential Suffering	Navigating fragility *Definition: The passive, baseline state of physical and existential vulnerability in the ICU*
Family support and affection Family unity Delegating decisions within the family Family pressure to undergo surgery	Family as a source of support and influence	Engaging Family in Advocacy *Definition: The interpersonal strategy of using the family as an emotional buffer and proxy*
Valuing nursing care (Half the cure) Humanizing care Being present and listening Trust in the doctor	Therapeutic environment and person-centred care	Managing Vulnerability through the Therapeutic Alliance *Definition: The clinical framework where person-centered nursing care mitigates patient suffering*
Faith and religious belief Fostering optimism and resilience Focusing on grand children and recreational activities Acceptance of the reality of the illness	Adjusting the Self to the oncological reality	Preserving Personhood through the Advocacy *Definition: The patient’s cognitive and spiritual adaptation to reframe identity and resilience*
Wanting to be informed and well-informed Clarification and justification An accurate recollection of events Overemphasis on details Navigating the healthcare system	Therapeutic empowerment	Synchronizing Advocacy with the Patient’s Self-Determination *Definition: The ethical alignment of nursing advocacy with the patient’s right to autonomy and information*
Omission of the word “cancer” Therapeutic optimism Denial or downplaying of the seriousness	Emotional defense and evasion	Shielding the Self *Definition: The active psychological defense mechanism of emotional evasion and denial to resist threat*

## Data Availability

The data is available upon request, but is not publicly and unconditionally accessible because this restriction is an ethical and legal requirement defined in our research protocol.

## Abbreviations

The following abbreviations are used in this manuscript:

GT	Grounded Theory
ICU	Intensive Care Unit
